# A mandatory modification in extracorporeal biventricular assist device (BIVAD) implantation: intercostal tunnel application: a case report

**DOI:** 10.1186/1749-8090-8-229

**Published:** 2013-12-15

**Authors:** Oztekin Oto, Gokhan Albayrak, Baran Ugurlu, Yusuf Kuserli, Ebru Ozpelit, Sema Guneri

**Affiliations:** 1 Department of Cardiovascular Surgery, Dokuz Eylul University, Mithatpasa Bulvari, Izmir, Turkey; 2 Department of Cardiovascular Surgery, Izmir University, Yeni Girne Bulvari. 1825 sok. No: 12 Karsiyaka, Izmir, Turkey; 3 Cardiovascular Surgery, Kahramanmaras Necip Fazil City Hospital, Yolu Kahramanmaras, Gaziantep, Turkey; 4 Department of Cardiology, Dokuz Eylul University, Mithatpasa Bulvari, Izmir, Turkey

**Keywords:** End stage heart failure, Heart transplantation, Ventricular assist device

## Abstract

In this case, our patient was a heart transplant candidate connected to a respiratory system. An extracorporeal biventricular assist device (BIVAD) was the only option in order to bridge to transplantation. In routine procedures, it is recommended that Berlin Heart Excor cannulas be removed through the subfascial subcostal tunnel. As the severely dilated right ventricle compressed the apex of the left ventricle, which was also dilated to the mid-back zone of the left hemithorax, the whole length of the Extracorporeal BIVAD apical cannula had to remain within the thorax; however, the cannula was removed from the body by creating a tunnel at the 7th intercostal space. In the long-term follow-up, this compulsory modification has proven to be safe and effective.

## Background

Today, heart transplantation in patients with severe heart failure can be performed worldwide, and its results have reached an encouraging level. The number of patients waiting for transplantation is increasing day by day; however, the shortage of donors still remains a common problem. The use of VAD as a bridge to transplantation, due to a shortage of donors, is expanding with the transition of these patients into the decompensated heart failure stage, resistant to pharmacological treatment [1]. In VAD patients, left ventricular (LV) inflow cannula is usually removed from the body with subcostal incisions through the costal margin, adjacent to the diaphragm. In patients with severely dilated LV, such as a dilated cardiomyopathy (DCM), removal of the LV inflow cannula from the body is difficult surgically [2]. In this article, we will present a case with DCM (New York Heart Association (NYHA) class V), who was a heart transplantation candidate requiring an emergency BIVAD as a bridge to transplantation. The main point of this letter is to demonstrate the safety and effectiveness of this modification in long term follow up.

## Case presentation

Our patient was a 51-year-old Caucasian male under follow-up with the diagnosis of New York Heart Association (NYHA) class V dilated cardiomyopathy. Two dimensional echocardiography revealed left ventrical ejection fraction (LVEF): 15%, right ventrical ejection fraction (RVEF): 20%, left ventricular dilatation, systolic diastolic dysfunction, moderate mitral valve insufficiency, severe tricuspid insufficiency, pulmonary artery pressure: 55 mmHg/24 mmHg and left atrial dilatation. Upon heart catheterization, the right atrial pressure and pulmonary artery pressure were 18 mmHg and 60/26 (43) mmHg, respectively. Pulmonary capillary wedge pressure was 21 mmHg. Calculated cardiac index was 1.8 L/min per square meter. Coronary arteriography was normal. According to the results of these investigations, heart transplantation was decided for the patient. The patient required treatment at the intensive care unit (ICU) as his general condition had deteriorated and an intra-aortic balloon pump was applied as a bridge to the next decision. During follow-up at the ICU, the patient suffered cardiac arrest. Despite intensive positive inotropic support and intra-aortic balloon pump support, systemic arterial pressure was 65/45 mmHg. Immediately afterwards, BIVAD implantation was decided for the patient.

**Figure 1:**
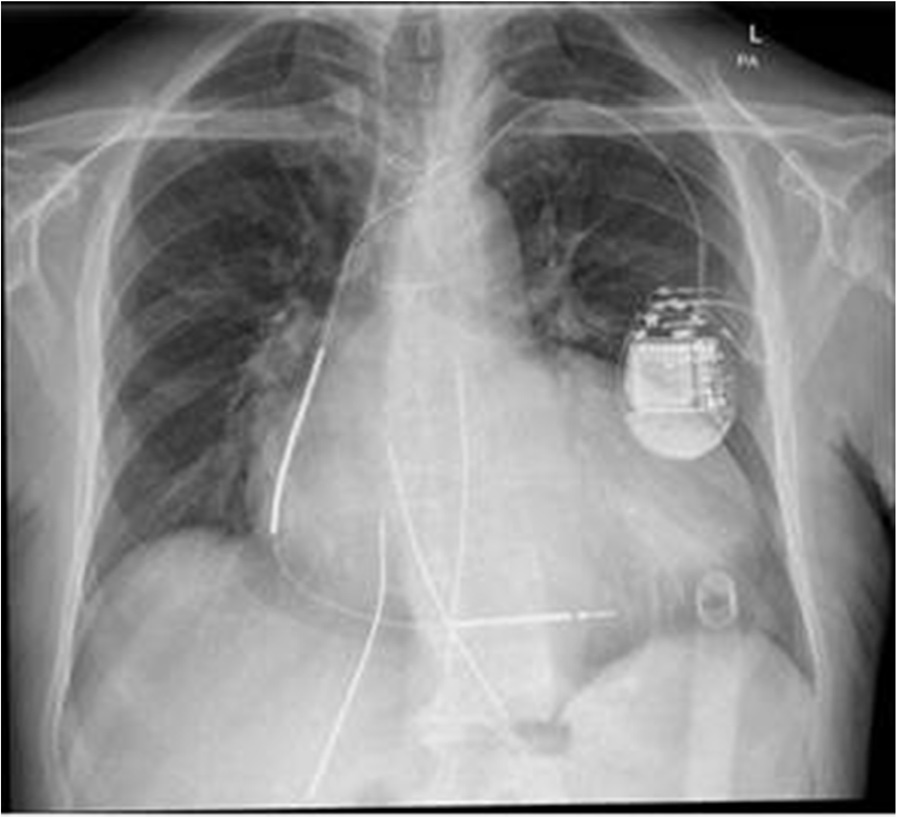
Chest X-ray of the patient on the first postoperative day.

**Figure 2:**
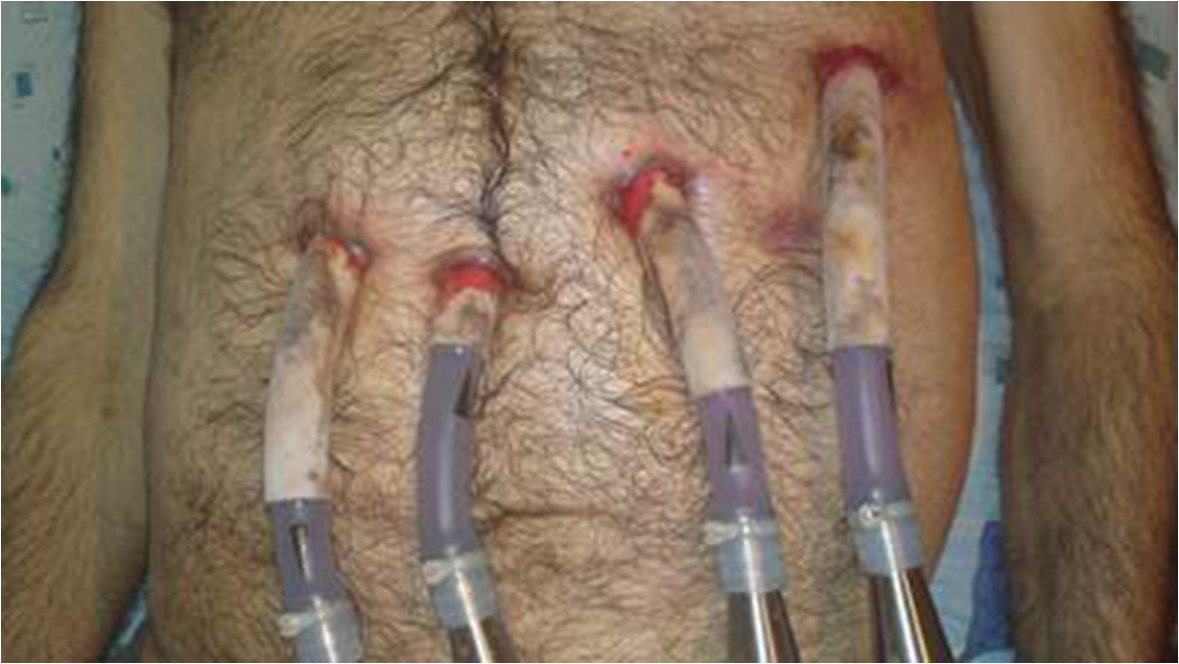
Positions of the cannulas on patient.

Under cardiopulmonary bypass (CPB), the apex cannula was placed first. Due to cardiomegaly and the long distance between the apex of the heart and the sternum, the apex cannula could not be removed from the anterior abdominal wall. The cannula, which was positioned on the left ventricular apex, was pushed to middle and back of the left hemithorax along the lateral edge of the left of the heart and was removed from the body through the extrapleural tunnel, placed in the 7th intercostal space (Figure 1). Cannulas of the right atrium, pulmonary artery, and ascending aorta were found and removed through the tunnel under the anterior abdominal wall (Figure 2). It was confirmed by computerized tomography angiography that the apex cannula, removed through the intercostal tunnel, was not kinked. Removal of the apex cannula from the intercostal space had no adverse effect on the success of BIVAD, and there was no pain due to compression of the intercostal nerves. The patient became more functional at the activities of daily life one week after BIVAD implantation.

Heart transplantation was performed one year after BIVAD implantation. Unfortunately, the patient died due to pneumonia and sepsis in the postoperative third month.

A written informed consent form was obtained from the patient for publication of this case report and accompanying images.

## Conclusion

**Figure 3:**
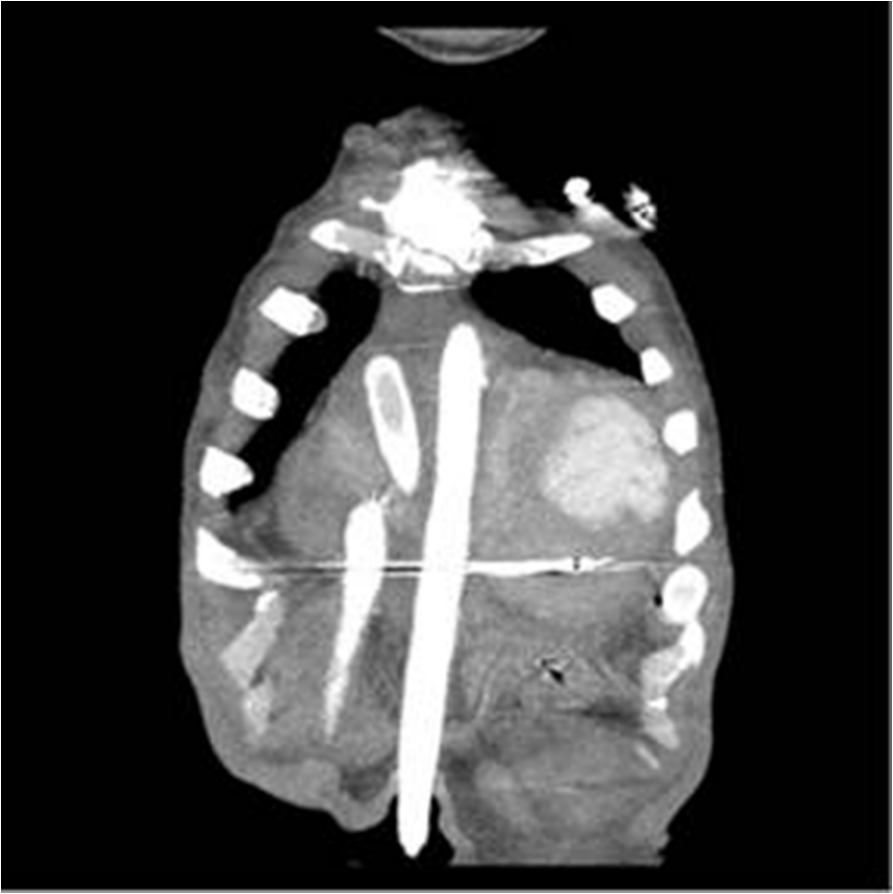
Appearance of the cannulas on CT angiography.

VAD implantation is a very well known procedure for advanced stage heart failure. The exact positioning is very important when inserting the left ventricular apex cannula. Since the cannula is placed very close to the interventricular septum or the papillary muscle, severe constraints on flow may ensue [3]. To prevent infection and pain, it is recommended that the Berlin Heart Excor cannulas be removed from the body through the subfascial tunnel [4]. In our case, it was predicted that the cannula could not be removed subcostaly, under the skin, because of corbovinum and the distance of the apex to the sternum. The apex cannula was removed through the intercostal space, and it was confirmed by computerized tomography angiography that the apex cannula was not kinked (Figure 3).

In BIVAD implantation, if the apex is not at the normal location due to cardiomegaly, and if coiling on the cannula is considered when the cannula is removed from subcostal region, the apex cannula can be removed through the intercostal space with a necessary modification.

## Consent

Written informed consent was obtained from the patient for publication of this Case report and any accompanying images. A copy of the written consent is available for review by Editor-in-Chief of this journal.

## Abbreviations

BIVAD: Biventricular assist device; VAD: Ventricular assist device; LV: Left ventricular; DCM: Dilated cardiomyopathy; NYHA: New York Heart Association; LVEF: Left ventricle ejection fraction; RVEF: Right ventricle ejection fraction; CPB: Cardiopulmonary bypass.

## Competing interests

The authors declare that they have no competing interests.

## Authors’ contributions

OO, GA, BU and YK performed the operation. They have contributions to conception, design, acquisition of data. EO and SG decided to implant ventricle assist device. All authors read and approved the final manuscript.

## References

[B1] Oosterom A de Jonge N Kirkels JH End-stage heart failure and mechanical circulatory support: feasibility of discharge from hospital Neth Heart J 2007 8 2 45 50 10.1007/BF03085953 17612659 PMC1847752

[B2] Drews T Loebe M Hennig E The ‘Berlin Heart’ assist device Perfusion 2000 8 387 396 10.1177/026765910001500417 10926425

[B3] Schmauss D Kaczmarek I Beiras-Fernandez A Tentacles: a novel device for exposing the heart for the insertion of left apical assist device cannulae Heart Surg Forum 2009 8 2 116 118 10.1532/HSF98.20091000 19383586

[B4] Hetzer R Potapov EV Stiller B Improvement in survival after mechanical circulatory support with pneumatic pulsatile ventricular assist devices in pediatric patients Ann Thorac Surg 2006 8 917 925 10.1016/j.athoracsur.2006.03.065 16928509

